# In Vitro Antifungal Activity of Silver Nanoparticles Biosynthesized with Beech Bark Extract

**DOI:** 10.3390/plants10102153

**Published:** 2021-10-11

**Authors:** Anca Delia Mare, Cristina Nicoleta Ciurea, Adrian Man, Mihai Mareș, Felicia Toma, Lavinia Berța, Corneliu Tanase

**Affiliations:** 1 Department of Microbiology, “George Emil Palade” University of Medicine, Pharmacy, Sciences and Technology of Târgu Mureș, 38 Gheorghe Marinescu Street, 540139 Târgu Mureș, Romania; anca.mare@umfst.ro (A.D.M.); felicia.toma@umfst.ro (F.T.); 2 Doctoral School, George Emil Palade University of Medicine, Pharmacy, Science, and Technology of Târgu Mureș, 38 Gheorghe Marinescu Street, 540139 Târgu Mureș, Romania; 3 Laboratory of Antimicrobial Chemotherapy, Ion Ionescu de la Brad University of Life Sciences, 8 Aleea Mihail Sadoveanu, 700489 Iași, Romania; mihaimares@fungi.ro; 4 Department of General and Inorganic Chemistry, “George Emil Palade” University of Medicine, Pharmacy, Sciences and Technology of Târgu Mureș, 38 Gheorghe Marinescu Street, 540139 Târgu Mureș, Romania; lavinia.berta@umfst.ro; 5 Department of Pharmaceutical Botany, “George Emil Palade” University of Medicine, Pharmacy, Sciences and Technology of Târgu Mureș, 38 Gheorghe Marinescu Street, 540139 Târgu Mureș, Romania; corneliu.tanase@umfst.ro

**Keywords:** silver nanoparticles, beech bark, antifungal activity, fluconazole synergism, biofilm inhibition, *ALS3*, *SAP2*, *HSP70*

## Abstract

Biosynthesis is a green method for the synthesis of silver nanoparticles (AgNPs). This study aimed to assess the antifungal activity of two silver nanoparticle solutions, synthesized using beech bark extract (BBE) and acetate and nitrate silver salts (AgNP Acetate BBE and AgNP Nitrate BBE), their influence on biofilm production, their potential synergistic effects with fluconazole, on different *Candida* spp., and their influence on virulence factors of *C*. *albicans* (germ tube production, gene expression for *ALS3*, *SAP2*, *HSP70*). Both the AgNP BBEs presented different minimum inhibitory concentrations for all the studied *Candida* spp., but biofilm production was inhibited only for *C*. *albicans* and *C*. *guilliermondii*. The growth rates of all the studied *Candida* spp. were inhibited in the presence of both AgNP BBEs, except for *C*. *auris*. Synergistic activity was observed for *C*. *parapsilosis* and *C*. *guilliermondii*, for different combinations of fluconazole with both the AgNP BBEs. The germ tube production of *C*. *albicans* was slightly inhibited by the AgNP BBEs. Only AgNP Acetate BBE was able to down-regulate the expression of *SAP2*. Overall, we can conclude that, even if more studies are necessary, AgNPs synthesized with beech bark extract might be an interesting alternative to classic antifungal treatments.

## 1. Introduction

*Candida* spp. are part of the commensal human mycobiota (skin, oral, genital, intestinal mucosa), but they are also recognized as important opportunistic pathogens that can promote mild to life-threatening infections, especially in immunocompromised patients. These infections, especially if acquired in the hospital, are associated with high rates of morbidity and mortality, with prolonged hospitalization, and they significantly increase healthcare costs. For *Candida* spp., the transition from a commensal microorganism to a pathogenic one is facilitated by various virulence factors (the adherence on tissue, invasion of the host cells, biofilm production, enzyme secretion, adaptability, etc.) [1,2,3,4,5]. Even if *C*. *albicans* is still considered the most important human fungal pathogen, in recent years, non-albicans *Candida* species (NAC), such as *C*. *parapsilosis*, *C*. *krusei*, *C*. *glabrata*, *C*. *auris*, etc., are more and more frequently identified as etiological agents of community and healthcare-associated infections, often with high transmissibility rates and limited options for treatment [6,7]. One of the most important causes that lead to the high colonization and infection rates with NAC is the widespread usage of antifungal agents, for treatment or prophylactic reasons [5,8].

The increasing emergence of multidrug-resistant *Candida* spp. (such as *C*. *auris*), along with less susceptible or intrinsically resistant species (such as *C*. *krusei*) and species that can develop acquired resistance to antifungal agents, is an important concern for the medical community [9,10,11]. This led to a new direction of research studies, focused on the development of different alternatives for classic antimicrobial agents. An important branch of these studies is focused on the synthesis, characterization, and biological activity of metal nanoparticles. Due to their shape and physical and chemical proprieties, metal nanoparticles are promising candidates for biomedical applications, drug delivery, or antimicrobial agents. Among these metal nanoparticles, silver nanoparticles (AgNPs) are the most studied ones because of their well-known antimicrobial (antibacterial, antifungal, antiviral), anti-inflammatory, and antioxidant activity and their low toxicity on animal cells [12,13,14,15,16]. Because the physical and chemical methods used for the synthesis of AgNPs are expensive, time-consuming, and with a negative impact on the environment, the latest studies focused on ”green” synthesis methods. Among these methods, the synthesis of AgNPs mediated by different parts of plant extracts (bark, leaves, fruit, seed, rhizome, flower, callus) is considered beneficial, even when compared with other biological methods, because it does not require aseptic conditions and cell cultures, and because of the possibility of the functionalization of different plant waste products [12,13,16,17,18]. Plant extracts contain a large variety of organic compounds (flavonoids, oils, alcohols, enzymes, quinines, phenolic and terpenoid compounds, etc.) and plant derivates (alginates, starch, chitin, cellulose, etc.) that can reduce metal ions in nanoparticles and act as stabilizing agents [19,20,21,22].

Along with the involvement of the plant’s components in the synthesis of AgNPs, the presence of the plant extract itself in the final solution of AgNPs is a great addition because of the well-known antimicrobial and antioxidant activity of medicinal plants’ extracts. Plant-derived biomolecules, such as flavonoids, quinones, terpenoids, saponins, tannins, steroids, glycosides, and alkaloids, are involved in the antimicrobial activity of plant extracts [23,24]. Multiple studies present not only the antibacterial effect of these plant compounds but also their antifungal activity, especially against *Candida* spp. [25,26,27,28,29].

In recent studies, the antifungal activity of different AgNPs was described, especially for *C*. *albicans*, but there is still a lack of information about their antifungal mechanisms and the effect on different virulence factors [30,31,32,33]. Previous studies [29,34] presented the biosynthesis of AgNPs using a polyphenolic beech bark extract and two silver salts, characterized these AgNPs, and demonstrated their antioxidant and antibacterial activity. The present study aims to further analyze the antifungal activity of these biosynthesized AgNPs against five different *Candida* spp., and to evaluate if these AgNPs might influence germ tube production (which facilitates adherence, the invasion process, and biofilm formation) and the expression of the *ALS3*, *SAP2*, and *HSP70* genes for *C*. *albicans*. *ALS3*, a surface protein of *C*. *albicans* hyphae, enables the yeast to attach to the surfaces of the cells and the biofilm extracellular matrix and to penetrate the host cell. *SAP*s genes encodes hydrolytic enzymes that facilitate the invasion of the host cell, while HSPs (heat shock proteins) enable yeast survival in hostile environments (low nutrients, high temperatures) [6].

## 2. Results

### 2.1. Silver Nanoparticle Synthesis

Beech bark extract and AgNP BBEs were previously synthesized and characterized, and the results of those studies (conducted by the same authors) were previously published. Briefly, the phytochemical evaluation of the aqueous BBE [35] demonstrated that the extract presented a total phenolic content of 69.76 GAE/g plant material, and identified the following compounds: gallic acid, vanillic acid, catechin, epicatechin, quercetin, protocatechuic acid, syringic acid, ferulic acid, chlorogenic acid, and isoquercitrin. Additionally, the mentioned study presented the antioxidant, antibacterial, antifungal, and antimutagenic potential of BBE. As the BBE characterization showed promising results, the next step of our research was the synthesis of silver nanoparticles using BBE. The results of this study were also previously published [34]. Briefly, the synthesis of AgNP BBEs was confirmed by the color change and by UV-Vis spectrometry (peak obtained at 420–475 nm). The Fourier transform infrared spectroscopy results confirmed that proteins and phenols from the BBE were mostly responsible for the stabilization and capping of the AgNP BBEs. Spherical or rarely triangular and polygonal particles (medium size of 32 nm) were described after the transmission electron microscopy examination of AgNP BBEs. Additionally, the AgNP BBEs presented antioxidant and antibacterial activity. Because of these promising previously published results, we considered it important to further characterize AgNP BBEs and to evaluate their antifungal activity.

### 2.2. The Antifungal Activity

As it is presented in Table 1, all the tested solutions exerted different degrees of inhibition against all the *Candida* spp. Beech bark extract alone did not exhibit inhibition against *C*. *albicans* at 50% concentration, but for both AgNP BBE solutions 50% and 100% inhibition were detected against *C*. *albicans*. In the case of *C*. *albicans* and *C*. *krusei*, the identified minimum inhibitory concentrations (MICs) for the AgNP BBEs were lower than those obtained for beech bark extract alone. For *C*. *albicans*, the AgNP BBE Nit MIC (100% inhibition) was lower than the one identified for AgNP Ac BBE (100% inhibition), but for *C*. *krusei* the MICs were similar for the AgNP BBEs (at 0.03 mg/mL—50% inhibition, and at 0.06–0.07 mg/mL—100% inhibition, respectively). For *C*. *auris*, the MICs of the AgNP BBEs were identified at the same dilution for both Ag NP BBEs and the beech bark extract, while in the case of *C*. *parapsilosis* and *C*. *guilliermondii*, the MICs for the AgNP BBEs were higher than the MIC identified for beech bark extract.

### 2.3. The Effect of Tested Solutions on Biofilm Formation

In most cases, at a concentration of 0.78%, the tested solutions inhibited biofilm formation to different degrees (Table 2). For *C*. *albicans* and *C*. *guilliermondii*, the beech bark extract alone presented significantly higher percentages of inhibition than both AgNP BBE solutions (*p* = 0.0001), but there were no significant differences between the percentages of inhibition for the AgNP Ac BBE and AgNP Nit BBE (very small differences were noticed, between 1 and 2%, *p* = 0.14). In the case of *C*. *auris*, the effect of the tested substances on biofilm production was indifferent, even if, overall, the tested substances presented a small degree of inhibition. The beech bark extract alone inhibited the production of biofilm for *C*. *parapsilosis*, and both AgNP BBE solutions highly stimulated the production of biofilm in this case. For *C*. *krusei*, both AgNP BBE solutions stimulated the production of biofilms, even if the effect of the beech bark extract alone was indifferent.

### 2.4. AgNP BBEs’ Effect on the Fungal Growth Rate

**Figure 1:**
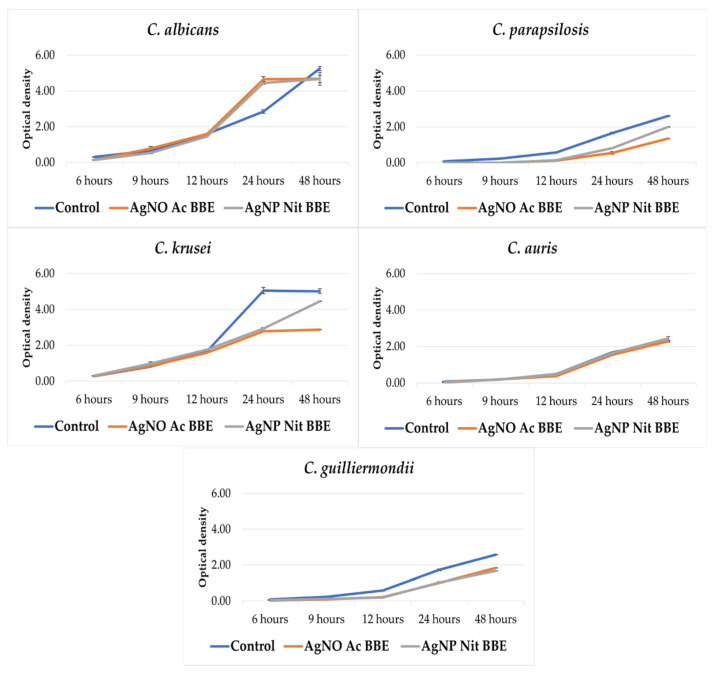
The effect of the AgNP BBEs on the growth rate of *Candida* spp.

Except for *C*. *auris*, the growth of all the other studied *Candida* spp. was inhibited in the presence of the AgNP BBEs (Figure 1). For *C*. *albicans* and *C*. *guilliermondii*, there was almost no difference between the growth in the presence of the AgNP Ac BBE and AgNP Nit BBE, while in the case of *C*. *parapsilosis* and *C*. *krusei*, the growth in the presence of AgNP Ac BBE was inhibited more than in the presence of AgNP Nit BBE.

### 2.5. Fluconazole Synergy Test—Checkerboard Method

**Figure 2:**
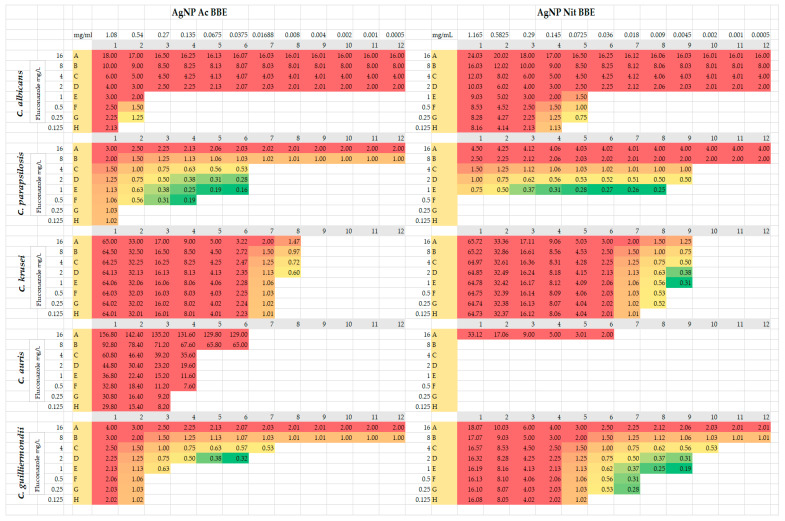
Checkerboard method—FIC results from synergy tests for AgNP BBEs in combination with fluconazole. The results highlighted in green (≤0.5) were considered synergistic, results highlighted in yellow (0.5–2) were considered indifferent, and results highlighted in red (>2) were considered antagonistic.

For *C*. *parapsilosis* and for *C*. *guilliermondii*, both AgNP BBE solutions presented synergistic activity with different concentrations of fluconazole (FIC values between 0.16 and 0.5) (Figure 2). In the case of *C*. *parapsilosis*, the combination of AgNP Ac BBE with fluconazole presented smaller FIC values (0.16, 0.19) than the combination of AgNP Nit BBE with fluconazole (FIC value 0.25). The FIC value of 0.16 was registered for the combination of 0.03 mg/mL AgNP Ac BBE with fluconazole 1 mg/L, while the 0.19 FIC value was registered for 0.06 mg/mL AgNP Ac BBE with fluconazole 1 mg/L and for 0.13 mg/mL AgNP Ac BBE with fluconazole 0.5 mg/L. The FIC value of 0.25 was registered for the combination of 0.009 mg/mL AgNP Nit BBE with fluconazole 1 mg/L. For *C*. *guilliermondii*, the combination of fluconazole with AgNP Nit BBE presented lower FIC values than the combination with AgNP Ac BBE (0.19 for 0.004 mg/mL AgNP Nit BBE with fluconazole 1 mg/L, and 0.32 for 0.03 mg/mL AgNP Ac BBE and fluconazole 2 mg/L, respectively).

For *C*. *krusei*, the combination of 0.004 mg/mL AgNP Nit BBE and fluconazole between 1 and 4 mg/L presented synergistic activity, but for the AgNP Ac BBE solution synergistic activity with fluconazole was not observed (the lowest FIC was 0.6, considered indifferent).

For *C*. *albicans* and *C*. *auris*, no synergistic activity was observed for fluconazole with both AgNP BBEs. For *C*. *albicans*, the lowest FIC (0.75, indifferent value) was in the case of 0.07 mg/mL AgNP Nit BBE in combination with fluconazole 0.25 mg/L. For *C*. *auris*, the lowest value of FIC (2, considered as an antagonistic effect) was noted for 0.03 mg/mL AgNP Nit BBE in combination with fluconazole 16 mg/L.

### 2.6. AgNP BBEs’ Effect on Germ Tube Production

**Figure 3:**
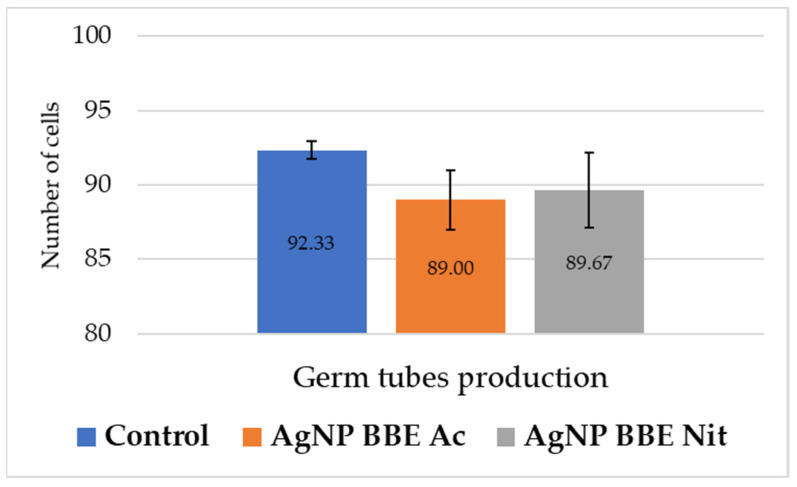
AgNP BBEs’ effect on *C*. *albicans* germ tube production.

As it is presented in Figure 3, both AgNP BBE solutions slightly reduced the formation of germ tubes for *C*. *albicans*, but no significant differences were registered (*p*-value between 0.3 and 1) compared to the control.

### 2.7. AgNP BBEs’ Influence on Gene Expression for ALS3, SAP2, and HSP70

**Figure 4:**
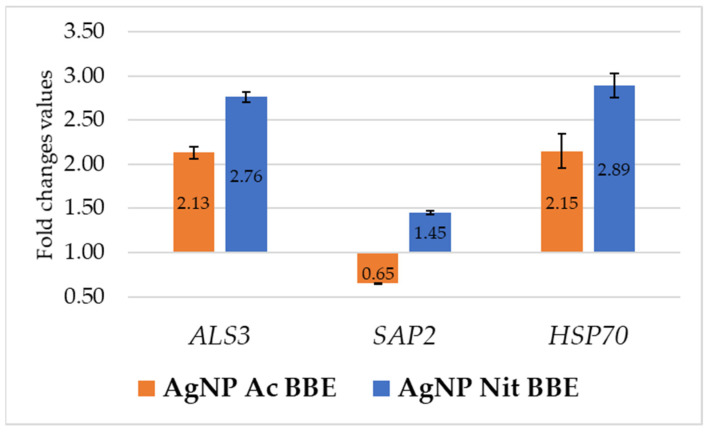
The influence of AgNP BBEs on *C*. *albicans* gene expression for *ALS3*, *SAP2*, and *HSP70*.

After 3 h of treatment with the identified MICs of AgNP BBEs, the expression of the *ALS3* and *HSP70* genes in *C*. *albicans* was up-regulated by both the AgNP BBE solutions. In both cases, the FC values for gene expression in the presence of AgNP Ac BBE (2.13 for *ALS3* and 2.15 for *HSP70*) were lower than those obtained in the presence of AgNP Nit BBE (2.76 for *ALS3* and 2.89 for *HSP70*). The *SAP2* gene expression was down-regulated only by the presence of AgNP Ac BBE (FC—0.65), while the treatment with AgNP Nit BBE up-regulated the *SAP2* gene expression, but with a smaller FC value than the one obtained for the expression of *ALS3* and *HSP70* in the presence of AgNP Nit BBE (Figure 4).

## 3. Discussion

The discovery of antimicrobial agents led to easy and efficient solutions for the treatment of infectious diseases, but, nowadays, because of the high toxicity of these agents, the increasing rates of multi-drug resistance strains, and the lack of new antimicrobial agents, “old” antimicrobial agents, such as silver or medicinal plants, are once again taken into consideration, at least as adjuvants for antimicrobial agents, and, in some cases, even as treatment options.

There is an abundance of information about the antibacterial activity and the antibacterial mechanisms of AgNPs [36,37,38,39], but studies about the antifungal activities of these solutions are still in their early stages. In recent studies, the antifungal activity of different AgNPs is described, especially for *C*. *albicans*, but there is still a lack of information about their antifungal mechanisms and the effect on different virulence factors [30,31,32,33]. Some of their possible antifungal mechanisms are the adhesion to the fungal membrane, reducing membrane fluidity, affecting the ergosterol and fatty acids levels, the production of ROS (reactive oxygen species), and DNA damage [33,40,41]. Along with these mechanisms, AgNPs synthesized with plants materials may have improved antifungal activity due to the biomolecules of the plant extracts.

In a recent study [35], the antifungal activity of beech bark aqueous extract was demonstrated against *C*. *albicans*, *C*. *parapsilosis*, and *C*. *zeylanoides*. This study reported similar MICs for *C*. *albicans* and *C*. *parapsilosis*, while our study identified higher MICs for *C*. *albicans* than for *C*. *parapsilosis*, differences that are probably generated by the different methodologies of the studies. A possible explanation of the antifungal activity of beech bark extract is its content of gallic, ferulic, protocatechuic, and syringic acids and phenolic compounds with proven antifungal activity [25,35]. An interesting observation of our study was that for *C*. *albicans* and *krusei* the AgNP BBEs presented lower MICs than the beech bark extract alone, for *C*. *auris* there was no difference, while for *C*. *parapsilosis* and *C*. *guilliermondii* the MICs for the AgNP BBEs were higher than the MICs for the beech bark extract alone.

Nanoparticles are considered a versatile option for the inhibition of biofilm productions, not only for bacterial ones, but also for *Candida* spp. biofilms, due to the small size of these nanoparticles, which allows them to easily penetrate the extracellular polymeric substance matrix. They may also act against biofilms by their intrinsic bioactivity or as nanocarriers (drug delivery systems) [42]. There are a few studies that present the inhibitory effect of AgNPs on biofilm formation, especially for *C*. *albicans* [43,44,45,46], with one study even indicating a dose-dependent inhibitory effect on biofilm formation and against pre-formed biofilms [47]. Another recent study [48] demonstrated dose-dependent and size-dependent antibiofilm activity for AgNPs, with the smaller size AgNPs (7.0 ± 1.6 nm) being more effective. In the mentioned study, higher doses of AgNPs were needed to inhibit biofilm formation for *C*. *albicans* than in the case of *C*. *parapsilosis*. In the present study, for *C*. *guilliermondii*, AgNP BBEs presented highest antibiofilm activity, followed by *C*. *albicans*. Even if the AgNP BBEs’ effect on *C*. *auris* biofilm was considered indifferent, a small degree of inhibition was observed. On the opposite side, for *C*. *parapsilosis* and *C*. *krusei,* the AgNP BBEs stimulated the production of biofilms, even if for *C*. *parapsilosis* the beech bark extract alone was able to inhibit it.

In this study, the growth rates of all the tested *Candida* spp., except for *C*. *auris*, were impaired by the MICs of both AgNP BBEs, especially for *C*. *guilliermondii* and *C*. *parapsilosis*. The AgNP Ac BBE was slightly more efficient than the AgNP Nit BBE in the case of *C*. *krusei* and *C*. *parapsilosis*. In a previous study, it was proven that AgNP BBEs present antibacterial effects against Gram-positive and Gram-negative bacterial strains, and the AgNP Ac BBE was able to inhibit the growth rate of *Staphylococcus aureus* and *Pseudomonas aeruginosa*, while *Escherichia coli* could not grow in the presence of these AgNP BBEs [34]. An interesting observation of the present study was that, for *C*. *albicans*, after 24 h, the growth rate was stimulated in the presence of the AgNP BBEs, compared to the control, but only for a short time, as after 48 h the growth rate was slightly inhibited compared to the control. A possible explanation comes from the great capacity of *C*. *albicans* to adapt and to respond to different environmental changes (pH, nutrition factors, temperature, etc.). *C*. *albicans* can adapt to low pH values (by remodeling their cell wall), as well to higher pH values (hyphal development) [49,50]. As the AgNP BBEs were alkaline solutions, the pH differences might explain the short time stimulation of the growth rate. To fully understand the antibacterial and antifungal mechanism of AgNPs, more research is needed, but the results from our studies prove, once again, that the biosynthesis of AgNPs with plant extracts can be considered a viable option in antimicrobial therapy.

In this study, synergic activity between different combinations of fluconazole with both the AgNP BBEs was observed for *C*. *parapsilosis* and *C*. *guilliermondii*, but for *C*. *albicans* and *C*. *auris* no synergistic activity was observed. Interestingly, for *C*. *krusei*, a strain intrinsically resistant to fluconazole, the combination of 0.004 mg/mL of AgNP Nit BBE presented synergistic activity with different concentrations of fluconazole (1–4 mg/L). Another study presented the decrease in the fluconazole MIC in the presence of biologically synthesized AgNPs for a fluconazole-resistant strain of *C*. *albicans* [51]. Even if the mechanisms of antifungal activity of AgNPs still need further studies, it was already demonstrated that AgNPs can disrupt the cell wall and the cytoplasmic membrane and can increase the production of hydroxyl radical and reactive oxygen species. The alteration of cell membrane permeability by AgNPs may be one factor that facilitates fluconazole entry into the cell, where it interferes with ergosterol biosynthesis [41,51,52].

Overall, in this study, the AgNP BBEs were able to present different degrees of biological activity against the studied *Candida* spp., except for *C*. *auris*. *C*. *auris*, known as a multidrug-resistant strain, exhibits high MICs for the AgNP BBEs, similar to the ones of the beech bark extract alone. At the studied concentration, the effect of the AgNP BBEs on biofilm production was minimal, the growth rate was not inhibited, and synergism with fluconazole was not detected. Other studies [47,53], with different methodologies, demonstrated that AgNPs can inhibit biofilm productions for *C*. *auris*. This emphasizes the fact that the antifungal activity of the different AgNPs is still in the early stages of research, especially for other strains than *C*. *albicans*, and more studies might bring significant benefits for understanding the utility of AgNPs in this field.

An important virulence factor of *C*. *albicans* is the morphological transitions to filamentous forms (hyphae, germ tubes). Morphological plasticity facilitates adherence, the invasion process, and biofilm formation [6,32]. In the present study, both AgNP BBE solutions slightly inhibited the formation of germ tubes, but no significant differences were registered compared to the control. Other studies presented high rates of germ tube inhibition, but for different methodologies and different types of AgNPs [32,54,55]. It was reported that, for *C*. *albicans*, biosynthesized AgNPs may interact with Ras-mediated signal transduction pathways by down-regulating the expression of the EGE1 gene (cell elongation gene), the *TUP1* and *RFG1* genes (hyphal transition genes), and the *TEC* gene (hyphal inducer gene) [32,56].

The effect of AgNP BBEs on gene expression for *C*. *albicans* was studied for three genes: *ALS3*, *SAP2*, and *HSP70*. *ALS3*, a surface protein of *C*. *albicans* hyphae, enables the yeast to attach to the surfaces of the cells and the biofilm extracellular matrix and to penetrate the host cell. *SAP*s genes encode hydrolytic enzymes that facilitate the invasion of the host cell, while HSPs (heat shock proteins) enable yeast survival in hostile environments (low nutrients, high temperatures) [6]. *ALS3* and *HSP70* gene expressions were up-regulated by a short treatment (of 3 h) with MICs of the AgNP BBEs, with higher values of FC in the case of AgNP Nit BBE than for AgNP Ac BBE. The *SAP2* expression was down-regulated only in the presence of AgNP Ac BBE. A few studies are available that present the influence of different nanoparticles on the expression of different genes of *C*. *albicans*, with different results, probably due to the different methodologies of the studies. A study presented that the expression of *ALS1*, *ALS3*, *HPW1*, and *EFG1* was down-regulated after 48 h of treatment with gold nanoparticles [57]. Another study observed the inhibition of *ALS1* and *ALS3* by zinc nanoparticles [58], while another study was able to prove that the time of incubation in the presence of zinc nanoparticles and the concentration influenced the down-regulation of the *SAP1*-*3* genes [59]. Another study proved that AgNP treatment of *C*. *albicans* down-regulated the *CDR2*, *ERG25*, *ERG1*, and *ERG11* expression, reduced the membrane fluidity and ergosterol levels, and produced elevated reactive oxygen species levels, confirming that AgNPs might be an alternative strategy for fluconazole-resistant *Candida* spp. [60].

## 4. Materials and Methods

### 4.1. Fungal Strains

The reference fungal strains used for the experiments were: *Candida albicans* ATCC (American Type Culture Collection) 90028, *Candida parapsilosis* ATCC 22019, *Candida krusei* ATCC 6258, *Candida auris* CBS (Fungal Biodiversity Centre) 10913, *Candida guilliermondii* 184 (the collection of the Cantacuzino National Research and Development Institute for Microbiology and Immunology, Bucharest). The strains are part of the collection from the Microbiology Department from UMPhST (George Emil Palade University of Medicine, Pharmacy, Science, and Technology of Târgu Mureş) and the Ion Ionescu de la Brad University of Life Sciences, Iași, stored at −70 °C. For each experiment, the strains were revitalized in Sabouraud Dextrose Broth (SDB, Oxoid, Basingstoke, United Kingdom) and incubated for 24–48 h, at 37 °C, in an orbital shaker.

### 4.2. Silver Nanoparticle Synthesis

Beech (*Fagus sylvatica* L.) bark extract and nanoparticles synthesis was performed according to previously described methods [22,29,34].

Briefly, 100 mL of distilled water were mixed with 10 g of beech bark and maintained for 30 min in an ultrasonic water bath, preheated at 70 °C (300 W heating power, 105 W, AC 150H, 40 KHz). The obtained solutions were filtered and filled up to 100 mL with distilled water.

For the AgNP biosynthesis, 10 mL of beech bark extract were mixed with 90 mL 1 mM silver acetate/nitrate solution. The pH of the obtained solutions was adjusted to 9 with NaOH. For the biosynthesis, the solutions were placed in an ultrasonic bath for 3 h, at 60 °C, until the color modification and the UV-Vis analyses suggested the biosynthesis of the nanoparticles. The obtained solutions were beech bark extract silver acetate, pH = 9 (AgNP Ac BBE), 2.16 mg/mL and beech bark extract silver nitrate, pH = 9 (AgNP Nit BBE), 2.33 mg/mL. The beech bark extract and the obtained nanoparticle solutions were sterilized with sterile syringe filters (0.45 µm pore diameter, Whatman, Kent, United Kingdom). Transmission electron microscopy analysis showed spherical, polygonal, and triangular particles with an average size of 32 nm [34].

### 4.3. The Antifungal Activity

The antifungal activity of the tested solutions was performed in 96-wells microtiter plates, according to the microdilution method. Two hundred microliters of the tested solutions were added in the wells of the first column of the microtiter plate, and 100 µL of RPMI 2X (RPMI-1640 Medium, Sigma-Aldrich, St. Louis, MO, USA), buffered with MOPS, in the rest of the microtiter plate; binary serial dilutions of the tested solutions were performed in RPMI. One milliliter from fungal 0.5 McFarland suspension was mixed with 9 mL RPMI to obtain an inoculum with a density of approximatively 1–5 × 10^5^ CFU/mL. One hundred microliters from this suspension were added to all the wells of the microtiter plate. Negative (RPMI medium) and positive (RPMI + fungal suspension) controls were also performed. The plates were incubated for 36 h at 37 °C. Fifty percent minimum inhibitory concentrations (MIC 50%) were considered in the wells where the fungal growth was reduced (visual examination) over 50% compared to the positive control, according to EUCAST recommendations, while 100% minimum inhibitory concentrations (MIC 100%) were considered in the wells where no fungal growth was observed.

### 4.4. The Effect of Tested Solutions on Biofilm Formation

The effect of tested solutions on *Candida* spp. biofilm production was studied in the microtiter plates where MIC was assessed. After the MIC was evaluated, the excess medium was removed, and the wells were washed twice, by immersing in sterile water, to clear away the unattached cells. Two hundred microliters of crystal violet 0.1%, aqueous solution, were added to each well, followed by a 15-min incubation at room temperature. Afterward, the plates were washed three times by immersing them in sterile water and left to dry for a few hours at room temperature. Two hundred microliters of 30% acetic acid were added to the wells and left for 15 min at room temperature to solubilize the crystal violet attached to the cells. The plates were shaken to homogenize the suspension and the absorbance was measured with a spectrophotometer at 620 nm, using as control a well that did not contain the tested substance (the positive control well). The effect of AgNP BBEs on biofilm formation was assessed in the wells where the concentration of the tested substance was 0.78% (0.01 mg/mL), as this was the maximum concentration where the growth of all the *Candida* spp. was not inhibited by the tested substances when the antifungal activity was studied. The influence of tested substances on the biofilm was calculated with the following formula:

$$100\times \frac{absorbance\;from\;the\;studied\;well}{absorbance\;from\;the\;control\;well}-100$$

The inhibition of the biofilm was considered when the results were negative and ≤−25%. If the results were between −25% and +25%, the effect was considered indifferent, while results ≥+25% were considered as the stimulation of biofilm production.

### 4.5. AgNP BBEs’ Effect on the Fungal Growth Rate

To evaluate the effect of the AgNP BBEs on the growth rate of *Candida* spp., the wells where MIC was identified in microtiter plates were reproduced in 10 mL volume, for each tested substance and species, and incubated for 48 h at 37 °C. A tube without the tested substances was used as growth control. At the initial moment and at 6, 9, 12, 24, and 48 h, 400 µL were removed and the optical density (OD) of the suspension was measured with a spectrophotometer, at 600 nm wavelength (Eppendorf BioPhotometer D30, Eppendorf, Wien, Austria).

### 4.6. Fluconazole Synergy Test—Checkerboard Method

The synergy tests were performed according to the checkerboard method [61,62], for each tested solution and each *Candida* strain. The checkerboard microplates were designed to include, on the same microtiter plate, all the EUCAST E.DEF 7.3.2 [63] clinical breakpoints for the tested strains (where available). One hundred microliters of two folded dilutions from the tested solutions were performed in each of the 8 rows of a 96-microtiter plate, in distilled water (on the first wells of each row, the final concentration of the solution was 50%; the last wells contained the lowest concentrations, 0.03%). Two folded dilutions of the fluconazole (Sigma-Aldrich) stock solution were prepared in RPMI 2X and 50 µL from the diluted solution were distributed in all the wells of each row of the microtiter plate (in the first row, the fluconazole final concentration was 16 µg/L in all twelve wells, in the second row the finale concentration was 8 µg/L, and in the 8th row, 0.125 µg/L). The fungal inoculum (0.5 McFarland) was diluted 1:10 in RPMI 2X and 50 µL from this suspension was distributed in each well of the microtiter plate. The plates were incubated for 36 h at 37 °C. Positive control well was considered the last well of the last row, the well where the fluconazole and the tested solution concentration were the lowest, while the negative control was considered the first well of the first row, where the fluconazole and the tested solution concentration were the highest. The fractional inhibitory concentration (FIC) was calculated with the following formula: FIC = FIC 1 + FIC 2, where FIC 1 was fluconazole MIC from the studied well/fluconazole MIC, and FIC 2 was the tested solution MIC from the studied well/tested solution MIC. The MIC for the fluconazole was considered the first well where fungal growth was 50% inhibited, at the lowest tested solution concentration, while the tested solution MIC was considered the first well where fungal growth was inhibited, at the lowest fluconazole concentration. If the FIC value was ≤0.5, the studied substances exerted a synergic effect with fluconazole. If the FIC value was between 0.5 and 2, the effect was considered indifferent, and if the FIC value was >2, the effect was considered antagonistic [63].

### 4.7. AgNP BBEs’ Effect on Germ Tube Production

To evaluate the effect of the AgNP BBEs on the germ tube production of *Candida albicans*, the tested substances’ MICs were reproduced in a 0.5 mL serum volume and mixed with 0.5 mL of 0.5 McFarland *C*. *albicans* suspension. A tube without the tested substance was used as the germ tube production control. The final suspension was incubated for two hours at 36 °C in a water bath. Afterward, wet mounts were performed using 25 µL from the prepared solutions and further examined by counting the number of germ tubes/100 cells.

### 4.8. AgNP BBEs’ Influence on Gene Expression for ALS3, SAP2, and HSP70

After incubating *C*. *albicans* in SDB in a shaking water bath overnight at 37 °C, a suspension of 2.1 AU (measured by OD at 600 nm wavelength) was obtained. From this suspension, 1 mL was centrifuged (5 min, 5000 rpm) in a sterile tube; the sediment was resuspended in a 1.5 mL volume of SDB mixed with the MIC concentration of the AgNP BBE solutions and incubated in a thermomixer for 3 h at 37 °C.

The extraction of the total RNA was performed by physical and chemical lysis followed by column purification. Briefly, the sediment, obtained after the centrifugation of the AgNP BBE-treated samples (5000 rpm, 5 min), was resuspended in 500 µL TE buffer (pH = 8). The cell wall was damaged by exposing the samples to two cycles of freezing (−70 °C) and heating (95 °C). The digestion of the cell wall was obtained by incubating the samples (60 min, 37 °C) with *Arthrobacter luteus* lyticase (Sigma-Aldrich, United States); afterward, the samples were heated (94 °C, 10 min) to neutralize the lyticase. From the obtained lysates, RNA purification was performed with silica-based spin columns (Quick-RNA Viral Kit, Zymo Research, Irvine, CA, United States). After RNA purification, one unit of DNase I solution (Thermo Scientific, United Kingdom) was used to remove the unwanted DNA from the elutes; the DNase was afterward inactivated (10 min, 65 °C). Then, nanodrop reading (Eppendorf BioPhotometer D30, Eppendorf, Wien, Austria) was used to assess the purity and the concentration of the extracted RNA (ng/µL).

After the extraction, reverse transcription was performed for each sample. GoScript Reverse Transcription System (Promega, Madison, WI, USA) was used to obtain complementary DNA from 1 µg purified RNA in a final 20 µL volume.

The RT-PCR assay was performed in MicroAmp optical 96-well reaction plate (Applied Biosystems, Waltham, MA, USA) using the QuantStudio 5 Real-Time PCR System (Thermo Fisher, Waltham, MA, USA) in a final volume of 20ul (GoTaq qPCR Master Mix, Promega). The primer sequences and the conditions used for amplification are presented in (Table 3). Finally, the expression levels of *ALS3*, *SAP2*, and *HSP70* were normalized to the ACT1 expression levels using the Delta–Delta Ct Method. If the fold change (FC) value was ˂0.75, the gene expression was considered down-regulated, and if it was >1.25, it was considered up-regulated.

### 4.9. Statistical Analysis

For the statistical analysis, Microsoft Office 365 and GraphPad QuickCalcs were used (significance value of *p* < 0.05). Tests were performed in triplicate, and the means of the results were taken into consideration.

## 5. Conclusions

Both AgNP Ac BBE and AgNP Nit BBE presented antifungal activity against all the studied *Candida* spp., with differences between the tested substances and strains. Biofilm production was inhibited only for *C*. *albicans* and *C*. *guilliermondii*. The growth rate of all the studied *Candida* spp. was inhibited in the presence of MICs of both AgNP BBEs, except for *C*. *auris,* whose MICs were very high and indicate a lack of activity. Synergistic activity was observed for *C*. *parapsilosis* and *C*. *guilliermondii* for different combinations of fluconazole with both the AgNP BBEs. Interestingly, for *C*. *krusei*, a strain intrinsically resistant to fluconazole, the combination of 0.0045 mg/mL of AgNP Nit BBE with fluconazole (1–4 mg/L) presented synergistic activity. The germ tube production of *C*. *albicans* was not significantly inhibited in the presence of the AgNP BBEs. Only AgNP Ac BBE was able to down-regulate the expression of *SAP2*. Overall, the tested AgNP BBEs presented different degrees of activity against all studied *Candida* spp., allowing us to conclude that AgNPs synthesized with beech bark extract might be an interesting alternative to the classic antifungal treatments.

## Figures and Tables

**Table 1:** Antifungal activity of beech bark extract and AgNP BBEs against *Candida* spp.

*Candida* spp.	Beech Bark Extract		AgNP Ac BBE				AgNP Nit BBE			
	MIC (50%) Inhibition	MIC (100%) Inhibition	MIC (50%) Inhibition		MIC (100%) Inhibition		MIC (50%) Inhibition		MIC (100%) Inhibition	
	%	%	%	mg/mL	%	mg/mL	%	mg/mL	%	mg/mL
*C*. *albicans*	>50%	>50%	6.25%	0.13	50%	1.08	6.25%	0.14	25%	0.58
*C*. *parapsilosis*	6.25%	>50%	50%	1.08	>50%	>1.08	50%	1.16	>50%	>1.16
*C*. *krusei*	6.25%	>50%	1.56%	0.03	3.13%	0.06	1.56%	0.03	3.13%	0.07
*C*. *auris*	6.25%	>50%	6.25%	0.13	>50%	>1.08	6.25%	0.14	>50%	>1.16
*C*. *guilliermondii*	3.13%	>50%	6.25%	0.13	>50%	>1.08	6.25%	0.14	>50%	>1.16

**Table 2:** The effect of tested solutions on biofilm formation.

*Candida* spp.	% of Biofilm Inhibition of Tested Substances		
	Beech Bark Extract	AgNP Ac BBE (0.01 mg/mL)	AgNP Nit BBE (0.01 mg/mL)
*C*. *albicans*	−46.79%	−32.11%	−33.94%
*C*. *parapsilosis*	−44%	92.44%	93.33%
*C*. *krusei*	15.09%	32.08%	52.83%
*C*. *auris*	−15.29%	−5.88%	−7.06%
*C*. *guilliermondii*	−90.87%	−83.04%	−84.13%

**Table 3:** Primer sequence conditions for RT-PCR.

Primers	Primer Sequences	Annealing Temperature
*ACT1* FW	5′–TTG TTG ACC GAA GCT CCA ATG–3′	60.6 °C
*ACT1* REV	5′–CCA CTT CAC AAT CCC CAT C–3′	
*ALS3* FW	5′–CCA CTT CAC AAT CCC CAT C–3′	56.0 °C
*ALS3* REV	5′–CAG CAG TAG TAG TAA CAG TAG TAG TTT CAT C–3′	
*SAP2* FW	5′–TAT TCA TAT GGC ATT ATT GGG TGG TA–3′	60.0 °C
*SAP2* REV	5′–AAC CAT TTC TGC TTG TTC TTC AGA–3′	
*HSP70* FW	5′–TGG TAT TCC ACC AGC TCC AAG–3′	61.4 °C
*HSP70* REV	5′–CAA CTT CTT CAA CAG TTG GTC CAC–3′	
